# 

**Published:** 1891-06

**Authors:** 


**Acute Rheumatism.**—As an external application.—*Med. Bulletin.*

℞ Salol.
Ætheris — parts, vi.
Callodii — parts, xxx.
M. —*London Medical Record.*

---

**For Acute Gastro-Enteritis for Children:**

℞ Creolin — gtt. iij.
Aq. Cinnamon — gtt. lxxv.
Syr. Marsh-mallow — gtt. xxij.

M. Sig.: Teaspoonful every hour for very young children.—*Schwinz.*

